# Density and electron density of aqueous cryoprotectant solutions at cryogenic temperatures for optimized cryoprotection and diffraction contrast

**DOI:** 10.1107/S2059798318003078

**Published:** 2018-04-27

**Authors:** Timothy J. Tyree, Ritwik Dan, Robert E. Thorne

**Affiliations:** a Cornell University, Ithaca, NY 14853, USA; b Physics Department, Cornell University, Ithaca, NY 14853, USA

**Keywords:** cryoprotectants, cryopreservation, aqueous glass, vitrification, thermal contraction, electron-density contrast, small-angle X-ray scattering, X-ray imaging, electron microscopy, cryocrystallography, cryogenic X-ray imaging, cryo-electron microscopy

## Abstract

The glass-phase densities at *T* = 77 K of aqueous solutions of the common cryoprotective agents (CPAs) methanol, ethanol, 2-propanol, glycerol, 2-methyl-2,4-pentanediol (MPD), ethylene glycol, polyethylene glycol 200 and polypropylene glycol 425 were measured as a function of CPA concentration. Individual drops with volumes as small as ∼65 pl were rapidly cooled to achieve the glass phase, and their densities at *T* = 77 K were determined by cryoflotation. These densities were used to determine the glass-phase electron density of each solution and its volume thermal contraction between room temperature and 77 K. When combined with data for the critical cooling rates required to achieve the glass phase *versus* CPA concentration, these yield alternative measures of cryoprotectant effectiveness. These reference data will aid in minimizing sample stresses and mechanical damage in cryocrystallography, in cryogenic temperature X-ray imaging and in vitrification-based cryopreservation protocols, and in maximizing electron-density contrast between cryoprotectant solutions and biomolecules in cryogenic temperature small-angle X-ray scattering experiments and cryo-electron microscopy.

## Abstract 2

The densities of aqueous solutions of eight common cryoprotectants were measured at *T* = 77 K and were used to determine electron densities at *T* = 77 K and thermal contractions on cooling from room temperature. The results provide a quantitative basis for choosing cryoprotectants to optimize outcomes in cryocrystallography, cryo-SAXS, cryogenic temperature X-ray imaging and vitrification-based biological cryopreservation.

## 1. Introduction   

The formation and physical properties of crystalline and glassy/vitreous/amorphous phases of water and of aqueous solutions are important in several areas of science and technology. In biological cryopreservation, ice crystals that form within cells can puncture membranes and damage other cellular components. Ice-crystal growth concentrates solutes in the remaining liquid, sometimes driving protein aggregation and/or denaturation (Fahy & Wowk, 2015 ▸). In biomolecular X-ray crystallography, the formation of internal and external ice damages crystals, increasing their mosaicity and reducing resolution (Rupp, 2009 ▸). In cryogenic temperature small-angle X-ray scattering (cryo-SAXS), large scattering at small wavevectors *q* (*i.e.* at small scattering angles 2θ) by even minute amounts of ice can overwhelm scattering from the biomolecule of interest (Meisburger *et al.*, 2013 ▸). Ice formation is also a critical problem in cryogenic temperature X-ray imaging of, for example, hydrated cells (Huang *et al.*, 2009 ▸; Rodriguez *et al.*, 2015 ▸; Lima *et al.*, 2009 ▸), and in cryo-electron microscopy (cryo-EM; Costello, 2006 ▸), especially in high-resolution single-particle cryo-EM, where diffraction images of enormous numbers of molecules must be combined to generate high-resolution structures. Even when crystalline ice does not form, thermal contraction or expansion on cooling to the glass phase can damage samples (Juers & Matthews, 2004 ▸; Kriminski *et al.*, 2002 ▸; Rabin *et al.*, 2006 ▸; Hopkins *et al.*, 2015 ▸). Differential contraction between internal and external solvent and protein crystals, between regions of a cell or tissue having different solvent contents, between cryo-SAXS samples and their holders, and between thin-film X-ray imaging and cryo-EM samples and their supports can cause sample deformation, creep, fracturing and microscale disorder. Solvent contraction or expansion on cooling also modulates the solvent electron density, and this may have large effects on the strength of the diffraction signal from biomolecules in cryo-SAXS and cryo-EM.

At cooling rates exceeding a critical cooling rate (CCR), glassy, vitreous or amorphous ice is obtained, having a crystalline ice volume fraction that is too small to measure (or smaller than a defined and measurable threshold, *e.g.* 1%). Critical cooling rates and ice formation can be reduced using cryoprotective agents (CPAs), which include monoalcohols, polyols, PEGs, salts and sugars. These compounds are soluble in water, make hydrogen bonds and have other interactions with water that interfere with its ability to form the open tetrahedrally coordinated network characteristic of crystalline hexagonal and cubic ice, lower the liquid–solid transition temperature and homogeneous nucleation temperature, modify the glass-transition temperature and inhibit the kinetics of ice nucleation and growth (Angell, 2002 ▸).

Many pure CPAs (*e.g.* glycerol) do not crystallize. The critical cooling rates of aqueous CPA solutions increase roughly exponentially with decreasing CPA concentration (Warkentin *et al.*, 2013 ▸). At CPA concentrations above ∼50%(*w*/*w*) critical cooling rates are generally <1 K s^−1^ and solutions can easily be vitrified in millilitre volumes, and so the vitrified densities can easily be determined (Alcorn & Juers, 2010 ▸). However, such large CPA concentrations can damage biological samples (for example owing to osmotic shock), change molecular conformations and displace weakly bound ligands that may be of interest in, for example, searches for new pharmaceutical compounds. As CPA concentrations decrease from 50 to 20%(*w*/*w*) – through the range relevant in, for example, cryocrystallography and single-cell cryo­preservation – the critical cooling rates increase from ∼1 to ∼10^4^ K s^−1^, and achieving these cooling rates can require the use of sub-microlitre sample volumes. Measuring cryogenic temperature glass-phase densities in this concentration range has thus been difficult, and for most cryoprotectants no data have been available for concentrations below 50%(*w*/*w*).

Here, we use a method based on cryoflotation (Loerting *et al.*, 2011 ▸) to determine the density and thermal contraction of solutions of eight common CPAs as a function of concentration. We calculate the resulting electron density and electron-density contrast with protein and nucleic acids relevant in cryogenic temperature X-ray and electron-diffraction measurements. We combine these data with data for critical cooling rates *versus* concentration to determine the thermal contraction and electron-density contrast *versus* critical cooling rate. These will facilitate the optimization of cryoprotection given constraints on sample size and on cooling method and rate.

## 2. Materials and methods   

Cryoprotectant solutions were prepared as described in the Supporting Information, giving typical concentration uncertainties of ∼1%(*w*/*w*) for methanol, ethanol and 2-propanol and <0.1%(*w*/*w*) for the less volatile CPAs.

Densities of vitrified aqueous cryoprotectant solutions at atmospheric pressure were measured using a method that we have described in detail elsewhere (Shen *et al.*, 2016 ▸, 2017 ▸). Our method is based on cryoflotation, in which samples are immersed in a liquid at cryogenic temperature, the density of the liquid is adjusted until the sample becomes neutrally buoyant, and the density of the cryogenic liquid is then determined using Archimedes’ principle by measuring the apparent weight of a test mass immersed in the liquid (Loerting *et al.*, 2011 ▸). To measure glass-phase densities to low CPA concentrations, the samples must be cooled rapidly, which requires the cooling of small-volume samples. In Loerting *et al.* (2011 ▸) pure water was aerosolized and sprayed onto a cryogenically cooled copper plate to build up a large (∼100 mg) sample. Samples built up from microdrops can have voids that reduce the apparent sample density. For aqueous solutions, evaporation during aerosolization can introduce large uncertainties in concentration. We instead dispense and project individual drops with volumes of ∼1 µl [for CPA concentrations greater than ∼50%(*w*/*w*)] to ∼65 pl (for the smallest CPA concentrations) onto a liquid nitrogen/argon surface at *T =* 77 K, where they are cooled at rates as high as ∼10^3^ K s^−1^.

Once a vitrified drop has been obtained, the density of the nitrogen/argon mixture is adjusted by adding nitrogen or argon until the drop is approximately neutrally buoyant. The density of the mixture is then measured by determining the apparent weight of a 1 g test mass immersed in the liquid. The smallest liquid density in which the drop floats and the largest density in which it sinks are then averaged to obtain an estimate of the drop density that is accurate to ±0.5%. Previously reported *T* = 77 K densities for several CPAs at 50%(*w*/*w*) (Alcorn & Juers, 2010 ▸), measured using large-volume samples, agree with the present values to within ±0.5%.

To ascertain whether individual drops are vitrified or not, we used a visual assay based on optical clarity (McFerrin & Snell, 2002 ▸; Chinte *et al.*, 2005 ▸), supported by X-ray diffraction and SAXS measurements (Berejnov *et al.*, 2006 ▸; Meisburger *et al.*, 2013 ▸). For a given cooling rate, drops generally show a transition *versus* CPA concentration from clear to milky/opaque over a narrow [∼2%(*w*/*w*)] concentration range, corresponding to a transition from a largely vitrified to a highly polycrystalline phase (Berejnov *et al.*, 2006 ▸; Meisburger *et al.*, 2013 ▸). As CPA concentrations are decreased from ∼50%(*w*/*w*) towards ∼10%(*w*/*w*), the cooling rates required for vitrification increase by a factor of 10^2^–10^3^ (Warkentin *et al.*, 2013 ▸) and the drop sizes needed to achieve these cooling rates decrease from ∼100 µm towards 1 µm (Kriminski *et al.*, 2003 ▸). We could not reliably determine the optical clarity for drops smaller than ∼40 µm, and this gave a CPA-dependent lower bound on the concentration range explored of 25–35%(*w*/*w*).

The optical assay also cannot easily distinguish fully vitrified drops from crystalline drops containing one or a few large ice crystals (and so having few scattering interfaces). Drops with large ice crystals are far more likely to be observed when using large drops that give slow cooling rates. Pure liquid glycerol, ethylene glycol (EG) and polyethylene glycol 200 (PEG 200) (Faucher *et al.*, 1966 ▸), and polypropylene glycol 425 (PPG 425; Johari *et al.*, 1988 ▸), are all good glass formers. All can be cooled into the glass phase using large (millilitre) sample volumes and much smaller cooling rates than were used here (∼1–10^4^ K s^−1^). The same should also be true of pure 2-methyl-2,4-pentanediol (MPD), although we have not found studies of the glass-forming properties of this compound. Of the monoalcohols, 2-propanol forms a glass even at very low cooling rates (≪0.1 K s^−1^; Ramos *et al.*, 2013 ▸) and ethanol vitrifies at cooling rates of <0.5 K s^−1^ (Haida *et al.*, 1977 ▸). However, pure methanol has only been vitrified by vapor deposition on a cryogenically cooled surface (Sugisaki *et al.*, 1968 ▸), suggesting a critical cooling rate comparable to or larger than those used here, and that the critical cooling rates of aqueous methanol solutions have a minimum at some intermediate concentration between ∼40 and 100%(*w*/*w*). We thus assume that clear drops are in all cases vitrified, except for those of aqueous methanol solutions at concentrations above ∼80%(*w*/*w*), where a milky/opaque to transparent transition *versus* drop size or concentration is not observed.

Densities ρ at 77 K were measured *versus* CPA concentration *x* [in (*w*/*w*)]. These densities and the corresponding room-temperature densities (obtained from prior work; Herráez & Belda, 2006 ▸; Bosart & Snoddy, 1928 ▸; Cristancho *et al.*, 2011 ▸; Rahbari-Sisakht *et al.*, 2003 ▸; Muller & Rasmussen, 1991 ▸; Zafarani-Moattar & Salabat, 1998 ▸) were fitted using fourthorder polynomials. The fit parameters for room temperature and 77 K are given in Tables 1 ▸ and 2 ▸, respectively.

Average electron densities ρ_e_ (in e^−^ Å^−3^) for each CPA solution were calculated using the measured solution density ρ (in g cm^−3^), the CPA concentration *x* in %(*w*/*w*), the number of electrons per molecule *Z* for each constituent of the solution and the molecular weight MW for each constituent as
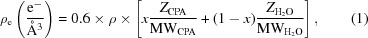
where the prefactor of 0.6 comes from the product of Avogadro’s number and the conversion from e^−^ cm^−3^ to e^−^ Å^−3^. Table 3 ▸ gives the values of *Z* and MW for each CPA and for water.

In small-angle X-ray scattering, the forward scattering *I*(*q*→0) = (Δρ)^2^ *V* ^2^, where Δρ (called the contrast) is the difference between the average electron density within the biomolecule’s envelope and the solvent that it displaces (Guinier & Fournet, 1955 ▸; Feigin & Svergun, 1987 ▸; Svergun & Koch, 2003 ▸). Adding cryoprotectants modifies the average solvent electron density and the density of the shell of perturbed solvent at the surface of the biomolecule, and thus modifies the forward scattering relative to that obtained in pure water. Ignoring changes in the perturbed solvent shell and the biomolecule volume *V*, the ratio of forward scattering for each CPA solution to that in pure water can be estimated as
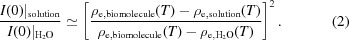
At room temperature ρ_e,protein_ = 0.43 e^−^ Å^−3^ (Crick, 1957 ▸) and 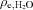 = 0.334 e^−^ Å^−3^, and at *T =* 77 K ρ_e,protein_ = 0.436 e^−^ Å^−3^ (assuming 1.3% volume contraction; Juers & Matthews, 2001 ▸) and 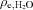 = 0.313 e^−^ Å^−3^ [assuming low-density amorphous (LDA) ice with ρ = 0.94 g cm^−3^]. Electron densities for nucleic acids are ∼0.55 e^−^ Å^−3^ (Svergun & Koch, 2003 ▸).

In general, the specific volume *v*_solution_ (in ml g^−1^) of a binary CPA solution with CPA weight fraction *x* will be different from the ‘ideal’ value calculated based on the specific volumes of its constituents in their pure form,
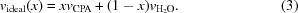
This difference gives the excess specific volume,
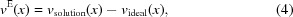
where *v*_solution_ = 1/ρ_solution_ at a particular CPA concentration. The excess specific volume provides a measure of the effects of interaction between the CPA and water.

## 3. Results   

### 3.1. Density and specific volumes   

**Figure 1:**
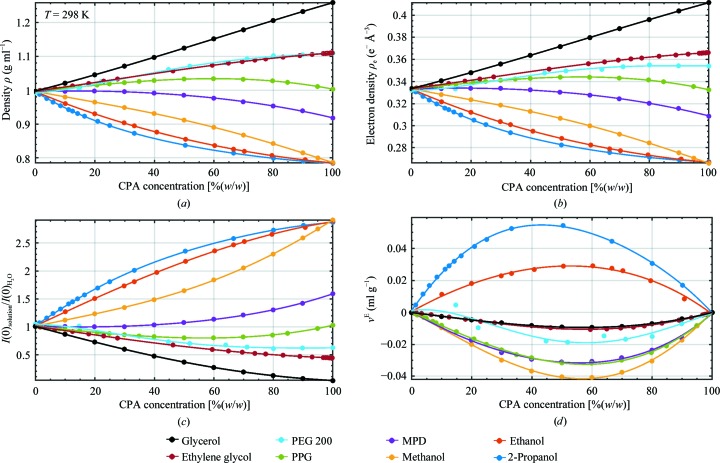
(*a*) Density ρ (g ml^−1^) *versus* CPA concentration [%(*w*/*w*)] near room temperature. Data for methanol, ethanol and 2-propanol (Herráez & Belda, 2006 ▸), glycerol (Bosart & Snoddy, 1928 ▸; Cristancho *et al.*, 2011 ▸), ethylene glycol and PEG 200 (Rahbari-Sisakht *et al.*, 2003 ▸; Muller & Rasmussen, 1991 ▸), and PPG 425 (Zafarani-Moattar & Salabat, 1998 ▸) were obtained from the cited literature. Data were measured at *T* = 298 K except for those for PEG 200, which were measured at 313 K. The solid lines are fourth-order polynomial fits with the coefficients given in Table 1 ▸. (*b*) Electron density (e^−^ Å^−3^) calculated from the densities in (*a*), equation (1) and Table 3 ▸. (*c*) Normalized forward scattering from protein *versus* CPA concentration, calculated using (2). The corresponding results for nucleic acids are shown in Supplementary Fig. S2(*a*). (*d*) Excess specific volume *v* ^E^ *versus* CPA concentration, calculated using (3) and (4) from the densities in (*a*).

Fig. 1 ▸ shows values for density (Fig. 1 ▸ *a*), electron density (equation 1; Fig. 1 ▸ *b*), normalized forward scattering (equation 2; Fig. 1 ▸ *c*) and excess specific volume (equation 3; Fig. 1 ▸ *d*) *versus* CPA concentration *x*, as determined from previously published density data at 298 K for all CPAs except PEG 200, which was measured at 313 K (Herráez & Belda, 2006 ▸). For all CPAs except PPG 425, the density varies monotonically with CPA concentration, and with generally modest deviations from linearity. Excess specific volumes *v* ^E^ have magnitudes below 0.05 ml g^−1^ or less than ∼5% of the measured specific volumes. Solutions of all CPAs except for ethanol and 2-propanol have larger densities ρ and smaller specific volumes *v* than are predicted using (3) based on the weight fractions and the densities of the pure CPAs. Methanol, ethanol and 2-propanol all have nearly identical *T* = 298 K densities in their pure form, but methanol solutions have substantially larger densities and large negative rather than positive excess specific volumes, reflecting the strong perturbation per unit CPA mass of the hydroxyl group on the largely tetrahedral hydrogen-bonding network responsible for the low density of water.

**Figure 2:**
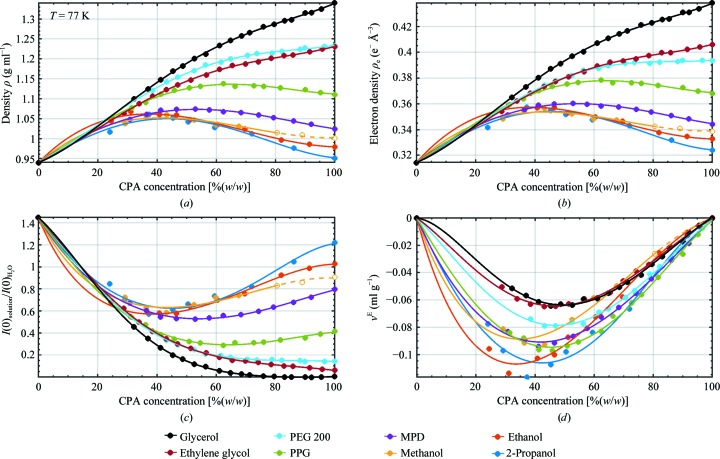
(*a*) Density ρ (g ml^−1^) *versus* CPA concentration [%(*w*/*w*)] at *T* = 77 K as measured here, with the accepted density of low-density amorphous (LDA) ice of 0.94 g cm^−3^ (Loerting *et al.*, 2011 ▸) plotted at *x* = 0. The solid lines are fourth-order polynomial fits with the coefficients given in Table 2 ▸. Density data for methanol solutions at concentrations above ∼80%(*w*/*w*), indicated by open circles, are likely to be for the α crystalline phase or a mixture of α and β crystal phases and possibly a vitreous component. Uncertainties in measured densities are dominated by the difference between the minimum nitrogen/argon mixture density at which a drop sinks and the maximum density at which it floats; the corresponding error bars are in most cases smaller than the symbols. (*b*) Electron density (e^−^ Å^−3^) calculated from the densities in (*a*), equation (1) and Table 3 ▸. (*c*) Normalized forward scattering for protein *versus* CPA concentration at *T* = 77 K, calculated using (2). Corresponding results for nucleic acids are shown in Supplementary Fig. S2(*b*). Normalization is by forward scattering in pure LDA ice at *T* = 77 K; Supplementary Fig. S1 normalizes by the forward scattering in pure water at *T* = 300 K. (*d*) Excess specific volume *v* ^E^ *versus* CPA concentration, calculated using (3) and (4) from the densities in (*a*). Values for methanol are based on the measured specific volume of pure methanol at 77 K, which is likely to be in a crystalline rather than a vitreous phase.

Fig. 2 ▸ shows results for density (Fig. 2 ▸ *a*), electron density (Fig. 2 ▸ *b*), normalized forward scattering (Fig. 2 ▸ *c*) and excess specific volume (Fig. 2 ▸ *d*) *versus* CPA concentration as determined here at *T* = 77 K, as well as values for pure LDA ice based on previous measurements (Loerting *et al.*, 2011 ▸). Electron-density contrasts for nucleic acids are shown in Supplementary Fig. S2. Unlike at room temperature, at 77 K the densities are nonmonotonic with concentration for all CPAs except glycerol, ethylene glycol and PEG 200 (which have the largest pure densities at 77 K), and all densities show large deviations from linearity. All solutions have larger densities and smaller specific volumes than are predicted using (4). The largest magnitude excess specific volume *v* ^E^ [for ethanol near 30%(*w*/*w*)] is roughly 12% of the measured specific volume *v*. All cryoprotectants thus appear to strongly disrupt the open bonded structure of pure low-density amorphous ice. The pure CPA densities at 77 K vary by a factor of 1.4, but at 30%(*w*/*w*) concentration the density variation has shrunk to a factor of only ∼1.04.

### 3.2. Thermal contraction   

**Figure 3:**
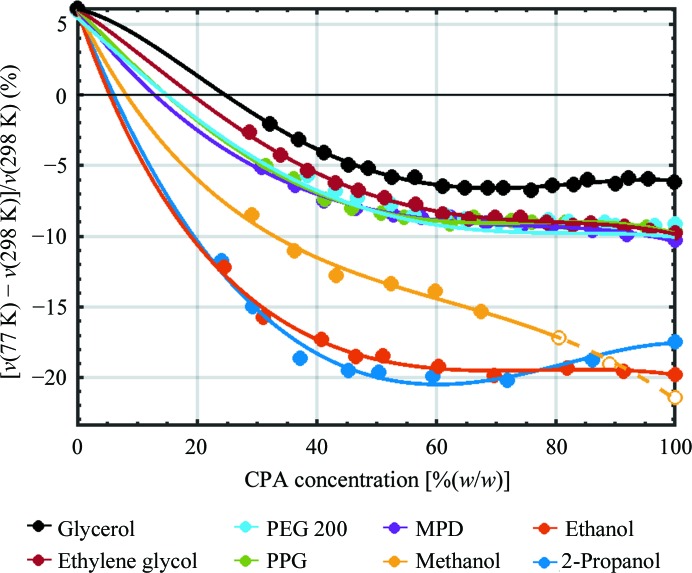
Volume contraction Δ*v*/*v* = [*v*(77 K) − *v*(298 K)]/*v*(298 K) in % for rapid cooling from room temperature to the glassy/vitreous/amorphous phase at *T =* 77 K *versus* CPA concentration [%(*w*/*w*)], determined from Figs. 1 ▸(*a*) and 2 ▸(*a*). Initial temperatures are *T* = 298 K except for PEG 200, for which data were only available at *T* = 313 K. Volume contractions for methanol solutions at 80%(*w*/*w*) and above are for cooling into a crystalline or mixed/crystalline amorphous phase and are indicated by open circles.

Fig. 3 ▸ shows the fractional change in specific volume [*v*(77 K) − *v*(298 K)]/*v*(298 K) on cooling from room temperature to *T* = 77 K. The monoalcohol solutions, which have the smallest room-temperature densities, show the largest thermal contractions; glycerol solutions, which have the largest room-temperature densities, show the smallest contractions. At 30%(*w*/*w*), contractions range from ∼15% for ethanol and 2-propanol and 9% for methanol to only 2% for glycerol solutions. Using the fits to extrapolate the measured densities to that of LDA ice, the CPA concentrations required to achieve no net contraction on cooling range from ∼6% for ethanol and 2-propanol and 8% for methanol to ∼25%(*w*/*w*) for glycerol.

### 3.3. Electron density and density contrast   

At room temperature, the solutions of the monoalcohols, especially ethanol and 2-propanol, provide the largest electron-density contrasts and glycerol the smallest. Monoalcohol solutions all have lower electron density and provide a larger contrast than pure water. However, they also have by far the largest thermal contractions on cooling. At *T* = 77 K, all CPA solutions have higher densities and electron densities and provide lower electron-density contrast at all concentrations than pure LDA ice. This is consistent with experience in single-particle cryo-electron microscopy, where even small amounts of cryoprotectants can produce an unacceptable loss of image contrast.

Previous cryo-SAXS measurements (Meisburger *et al.*, 2013 ▸; Hopkins *et al.*, 2015 ▸) used 45%(*w*/*w*) PEG 200 and 36%(*w*/*w*) propylene glycol solutions to prevent ice formation. At 40%(*w*/*w*), the monoalcohols give the largest electron-density contrast Δρ, and forward scattering *I*(*q*→0) ∝ (Δρ)^2^ roughly double that of a 40%(*w*/*w*) glycerol solution. The forward scattering in Fig. 2 ▸(*c*) is normalized by that obtained in LDA ice. A perhaps more useful comparison in cryo-SAXS is with the forward scattering at room temperature for molecules in (largely) CPA-free solvent having a density near 1; this indicates how background-subtracted cryo-SAXS and room-temperature SAXS signals will compare. Supplementary Fig. S1 shows the ratio of forward scattering in the CPA solution at *T* = 77 K to that in pure water at room temperature. The achievable forward scattering, assuming that CPA concentrations of 30–45%(*w*/*w*) are needed to prevent ice formation, is between ∼60 and 70% of that obtained at room temperature.

At concentrations below ∼20%(*w*/*w*), extrapolations between our data and data for LDA ice suggest that all CPAs will give comparable densities, electron densities and forward scattering. Selection of a CPA in this concentration range can then be based on effectiveness in suppressing ice formation, thermal contraction and effects (for example conformation changes and aggregation) on the biomolecule being studied.

## 4. Discussion   

### 4.1. Optimizing cryoprotectant choice   

Previous studies have determined the minimum cooling rates, termed critical cooling rates (CCRs), that are required to obtain ice-free, glassy samples *versus* CPA concentration for several cryoprotectants, for cooling rates between ∼1 and 10 000 K s^−1^ (Warkentin *et al.*, 2008 ▸, 2013 ▸). These data indicate that critical cooling rates vary exponentially with CPA concentration (proportional to the CPA number density) as
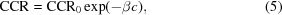
where CCR_0_ is the critical cooling rate for pure water (taken as 3 × 10^5^ K s^−1^), *c* is the CPA concentration in %(*w*/*v*) [not %(*w*/*w*)] at room temperature and β is a CPA-dependent constant. This has been explained using classical nucleation theory by assuming that ice formation during rapid cooling is nucleation-dominated and that solutes must be excluded from a critical nucleus (Warkentin *et al.*, 2013 ▸). Supplementary Table S1 gives the values of β for methanol, ethanol, ethylene glycol, glycerol and PEG 200 obtained from CCR data. Supplementary Fig. S2 plots these fits *versus* CPA concentrations in %(*w*/*w*) instead of %(*w*/*v*), using previous data for room-temperature solution densities (shown in Fig. 1 ▸ *a*) for the conversion. On a *w*/*w* basis, ethanol and methanol give the smallest CCRs, and glycerol, ethylene glycol and PEG 200 are comparably effective. At 30%(*w*/*w*) the CCRs for methanol, ethanol, ethylene glycol, glycerol and PEG 200 are ∼50, ∼6, ∼485, ∼320 and ∼250 K s^−1^, respectively.

In cryopreservation, cryocrystallography and cryo-SAXS, the maximum achievable sample-cooling rate is determined by the sample size and cooling method. Consequently, one often wants to choose a CPA that optimizes the properties of the sample when it is cooled at that maximum rate. Supplementary Fig. S3 combines fits for critical cooling rate *versus* CPA concentration in Supplementary Fig. S2 with the present density fits to obtain the relations between density and electron density at *T* = 77 K and the critical cooling rate.

**Figure 4:**
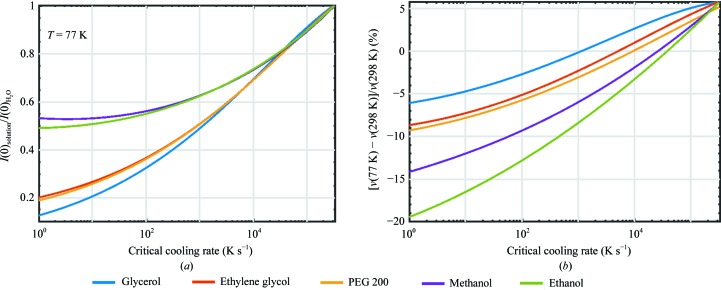
(*a*) Normalized forward scattering at *T* = 77 K *versus* CPA solution critical cooling rate (CCR) as determined from fits to the data in Fig. 2 ▸(*c*) and fits to the critical cooling rate (CCR) *versus* CPA concentration data (Warkentin *et al.*, 2013 ▸) shown in Supplementary Fig. S3, given by (5) with parameters β given in Supplementary Table S1. The CPA concentrations corresponding to critical cooling rates of 1 K s^−1^ range from ∼36%(*w*/*w*) for ethanol to ∼57%(*w*/*w*) for ethylene glycol. Critical cooling rate *versus* concentration data are not available for 2-propanol, MPD and polypropylene glycol. (*b*) Volume contraction Δ*v*/*v* = [*v*(77 K) − *v*(298 K)]/*v*(298 K) in % for rapid cooling from room temperature to the glassy/vitreous/amorphous phase *versus* the critical cooling rate of the CPA solution.

Fig. 4 ▸(*a*) shows the forward scattering *I*(*q*→0) ∝ (Δρ)^2^ for protein in CPA solution at *T* = 77 K normalized by that in LDA ice, as in Fig. 2 ▸(*b*), *versus* critical cooling rate. Glycerol gives the smallest contrast and methanol the largest at all CCRs. For a typical cooling rate in crystallo­graphy and cryo-SAXS of ∼500 K s^−1^ (Teng & Moffat, 1998 ▸; Walker *et al.*, 1998 ▸; Warkentin *et al.*, 2006 ▸), the normalized forward scattering is 0.43 for glycerol and 0.6 for methanol.

Fig. 4 ▸(*b*) shows the thermal contraction between room temperature and 77 K *versus* the critical cooling rate. For a given CCR, glycerol gives the smallest change in specific volume and ethanol the largest. For a CCR of ∼500 K s^−1^, the critical concentration of glycerol gives 1% contraction and the critical concentration of ethanol gives almost 10% contraction.

Cryoprotectant choice is also dictated by effects on protein stability and aggregation. Large monoalcohol concentrations can be destabilizing, while glycerol is typically stabilizing. For proteins in solution, monoalcohols can usually be tolerated at low concentration (requiring fast cooling) or at higher concentrations in combination with a stabilizing CPA or by adjusting, for example, the pH (Douzou, 1971 ▸; Wang *et al.*, 2015 ▸; Travers & Barman, 1995 ▸). In protein crystals, monoalcohols have frequently been used as cryoprotectants (Douzou *et al.*, 1975 ▸; Tilton *et al.*, 1992 ▸), including methanol at concentrations as high as 65%(*w*/*w*) (Singh *et al.*, 1980 ▸) and ethanol at concentrations as high as 85%(*w*/*w*) (Petsko, 1975 ▸), without obvious deleterious effects on protein structure. This in part reflects the often enormously stabilizing influence of the crystalline environment.

### 4.2. Cooling rates and thermal contraction   

Figs. 4 ▸(*a*) and 4 ▸(*b*) indicate that increasing the cooling rates from 500 to ∼20 000 K s^−1^, which is achievable in crystallo­graphy and cryo-SAXS using suitable cooling methods, sample sizes and sample holders (Warkentin *et al.*, 2006 ▸; Pflugrath, 2015 ▸), would allow cryogenic temperature electron-density contrast for all CPA solutions to be increased towards the value provided by LDA ice, and would reduce changes in the specific volume on cooling for all CPAs except glycerol. However, during cooling, differences in contraction between the protein, solvent and unit cell drive solvent redistribution within the crystal (as occurs, for example, with a wet sponge when it is squeezed), transferring it to or from the crystal surface and/or to or from disordered regions with higher or lower solvent concentrations that may form within the crystal and that are a likely cause of increased crystal mosaicity at cryogenic temperatures (Juers & Matthews, 2001 ▸; Kriminski *et al.*, 2002 ▸). Cooling slowly (without ice nucleation) may allow long-range solvent redistribution to occur (for example to the crystal surface or to a small number of disordered regions) without appreciably disrupting crystalline order, even when solvent and crystal contraction mismatches are large (Warkentin & Thorne, 2009 ▸). Large cooling rates reduce the time available for solvent redistribution, and for a given solvent composition are likely to generate a larger density of disordered crystal regions than would slower cooling. This effect of cooling rate may offset some of the gain owing to the use of smaller CPA concentrations that give smaller overall specific volume changes that faster cooling allows.

In cryo-electron microscopy, some sample motion may be owing to beam-induced relaxation of sample stresses associated with differential contraction between the sample (typically cryoprotectant-free buffer, the volume of which increases by ∼6%) and the supporting film and grid (the linear dimensions of which contract by perhaps 0.2%) during cooling. Sample stresses are most likely to be dominated by contraction during cooling from the glass-transition temperature of the sample (∼140 K) to the measurement temperature. Fig. 3 ▸ suggests that solutions containing ∼5%(*w*/*w*) ethanol or 2-propanol may eliminate the specific volume change of the sample, with a reduction in the electron-density difference between solvent and protein at cryogenic temperature of a modest 5%.

### 4.3. Glass-phase density measurements at smaller CPA concentrations   

The present glass-phase density measurements extend to minimum CPA concentrations of 25–35%(*w*/*w*). Based on the data in Fig. 2 ▸(*a*), density estimates at lower CPA concentrations obtained from fits that include the density of pure LDA ice should be accurate to roughly ±2%. Obtaining glass-phase densities at CPA concentrations between ∼10 and 25%(*w*/*w*) is technically quite challenging but is likely to be feasible. Larger cooling rates (∼10^3^–10^5^ K s^−1^) are required, and these could be obtained using smaller (∼1–20 µm) drops and also by cooling the drops in liquid propane/ethane before transfer to the nitrogen/argon mixture for density measurements. For such small drops, accurate assessment of their state (crystalline or vitreous) will require X-ray diffraction or SAXS measurements, performed before or after density measurements, and handling from drop creation through diffraction and density measurements in a way that eliminates all sources of ice contamination.

### 4.4. Effects of protein on the critical cooling rates and thermal contraction of CPA solutions   

In the nanoconfined environment of protein (or nucleic acid) crystals and in dehydrated biological samples, inter­action between solvent and protein strongly modifies the nucleation and crystallization behavior of the solvent (Warkentin & Thorne, 2009 ▸; Sartor *et al.*, 1995 ▸), properties that are determined by kinetics, and may also modify temperature-dependent average solvent densities (Svergun *et al.*, 1998 ▸). However, the presence of proteins at concentrations of interest in cryo-SAXS and cryoelectron microscopy (∼1–100 mg ml^−1^), and perhaps even in biological cryopreservation of fully hydrated cells and tissues, is likely to have little effect on solvent properties at CPA concentrations above ∼10%(*w*/*w*). Proteins are poor cryoprotectants on a *w*/*w* basis: we find that the critical cooling rates for 50%(*w*/*w*) lysozyme solutions are in excess of 10^4^ K s^−1^ (Hopkins *et al.*, 2012 ▸), consistent with results for hydrated protein powders (Sartor *et al.*, 1995 ▸). Proteins are less effective because only a fraction of protein atoms are solvent-exposed, because for a given *w*/*w* fraction large protein-free solvent clusters are more probable than large CPA-free clusters, and perhaps also because solvent-exposed protein atoms are on average less disruptive to bulk water structure than are those of common CPAs.

## 5. Conclusions   

The present results provide quantitative data and fits for optimizing cryoprotectant choice and concentration in cryocrystallography, cryo-SAXS, cryogenic temperature X-ray imaging and vitrification-based protocols for single-cell cryopreservation, given constraints on cooling rates, sample thermal contraction and/or electron-density contrast between biomolecules and the solvent.

The cryoprotectants studied here include those known to be most effective on a *w*/*w* basis in vitrification/fast-cooling protocols, where ice formation tends to be dominated by nucleation rather than growth, and those with low electron densities per unit volume. Sugars (glucose, sucrose and trehalose) are less effective in inhibiting ice nucleation on a *w*/*w* basis and their solutions have high electron densities at a given concentration in %(*w*/*w*) [∼0.41 e^−^ Å^−3^ for 50%(*w*/*w*) at 77 K, based on the data in Alcorn & Juers (2010 ▸)]. DMSO and salts (Rubinson *et al.*, 2000 ▸), with the exception of lithium acetate, will give higher electron densities and lower electron-density contrast at cryogenic temperatures than the CPAs studied here, as they do at room temperature. Both sugars and salts are used in contrast-matching experiments (Jeffries *et al.*, 2016 ▸), which may be easier/require lower concentrations at cryogenic temperatures owing to the excess thermal contraction of solutions relative to protein in the relevant CPA concentration range.

Ternary and higher order CPA mixtures, while common in conventional (large sample, low cooling rate) cryopreservation (Fahy & Wowk, 2015 ▸) and included in commercial cryoprotectant screens used in cryocrystallography, have received limited quantitative study in the vitrification (fast-cooling) regime (Garman & Mitchell, 1996 ▸; McFerrin & Snell, 2002 ▸; Chinte *et al.*, 2005 ▸). These are worthy of further exploration.

Aside from their utility in minimizing thermal stresses and maximizing electron-density contrast, the present results can be used to estimate the density of bulk-like solvent within cryocooled protein crystals and provide a check or constraint on solvent densities obtained in crystallographic refinement. Deviations of refined densities from the present values can also be used to assess how solvent structure and solute concentrations may deviate from bulk values within the hydration layers of the crystals.

## Supplementary Material

Additional experimental details and additional figures that plot the data in other, useful ways. . DOI: 10.1107/S2059798318003078/di5016sup1.pdf

## Figures and Tables

**Table 1:** Parameters of fourth-order polynomial fits ρ(*x*) = *a*_0_ + *a*_1_ *x* + *a*_2_ *x* ^2^ + *a*_3_ *x* ^3^ + *a*_4_ *x* ^4^ to the densities of aqueous CPA solutions at *T =* 298 K (except for PEG 200, which was measured at *T* = 313 K) *versus* CPA concentration *x* in %(*w*/*w*) for previously published density data shown in Fig. 1 ▸(*a*) The root-mean-square error (RMSE) and adjusted 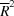 value are given for each fit.

Cryoprotectant	*a*_4_	*a*_3_	*a*_2_	*a*_1_	*a*_0_	RMSE	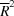
PPG 425	0.0043	−0.0977	0.0084	0.0916	0.9967	0.00031	0.9993
PEG 200	0.2307	−0.5680	0.3795	0.0767	0.9911	0.00295	0.9906
MPD	−0.0217	0.0125	−0.0914	0.0227	0.9965	0.00069	0.9988
Ethanol	0.0341	−0.0990	0.2267	−0.3726	0.9965	0.00059	0.9999
2-Propanol	0.1570	−0.4721	0.6405	−0.5364	0.9960	0.00471	0.9999
Glycerol	−0.0114	−0.0154	0.0578	0.2299	0.9971	0.00007	1.0000
Ethylene glycol	0.0173	−0.0778	0.0532	0.1202	0.9971	0.00009	1.0000
Methanol	0.0907	−0.2152	0.0832	−0.1688	0.9969	0.00034	1.0000

**Table 2:** Parameters of fourth-order polynomial fits ρ(*x*) = *a*_0_ + *a*_1_ *x* + *a*_2_ *x* ^2^ + *a*_3_ *x* ^3^ + *a*_4_ *x* ^4^ to the density of aqueous CPA solutions in their vitreous/amorphous phase at *T* = 77 K *versus* CPA concentration *x* in %(*w*/*w*) The root-mean-square error (RMSE) and adjusted 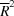 value is given for each fit.

Cryoprotectant	*a*_4_	*a*_3_	*a*_2_	*a*_1_	*a*_0_	RMSE	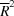
PPG 425	0.5992	−1.1164	0.2291	0.4605	0.9398	0.00231	0.9965
PEG 200	0.6787	−1.4281	0.6472	0.3962	0.9400	0.00161	0.9993
MPD	0.2085	−0.1907	−0.4414	0.5085	0.9398	0.00169	0.9963
Ethanol	−0.0663	0.7349	−1.3690	0.7389	0.9399	0.00469	0.9755
2-Propanol	0.8019	−1.2588	0.0597	0.4107	0.9890	0.00744	0.9428
Glycerol	0.7988	−1.7791	1.0744	0.3050	0.9400	0.00167	0.9996
Ethylene glycol	0.6169	−1.2632	0.5814	0.3559	0.9399	0.00152	0.9994
Methanol	0.0549	0.3903	−0.9959	0.6134	0.9396	0.00410	0.9726

**Table 3:** Electrons per molecule *Z* and molar mass MW of pure CPAs and water used in equation (1) to convert mass density into electron density

Substance	MW (g mol^−1^)	*Z* (e^−^ per molecule)
Water	18	10
Methanol (CH_4_O)	32.04	18
Ethanol (C_2_H_6_O)	46.07	26
2-Propanol (C_3_H_8_O)	60.10	34
Ethylene glycol (C_2_H_6_O_2_)	62.07	34
Glycerol (C_3_H_8_O_3_)	92.09	50
MPD (C_6_H_14_O_2_)	118.18	66
PEG 200 (C_2*n*_H_4*n*+2_O_*n*+1_)	200 (*n* = 4.13)	109.2
PPG 425 (C_3*n*+1_H_6*n*+2_O_*n*+1_)	425 (*n* = 6.80)	233.6
