# Supplementary material for: Density and electron density of aqueous cryoprotectant solutions at cryogenic temperatures for optimized cryoprotection and diffraction contrast
Source: Acta Crystallogr D Struct Biol. 2018 Apr 27;74(Pt 5):471–9. doi: 10.1107/S2059798318003078 (PMC5930352; doi:10.1107/S2059798318003078)
Supplement: Supplementary file 1 [file d-74-00471-sup1.pdf]

STRUCTURAL BIOLOGY

Volume 74 (2018)

Supporting information for article:

Density and electron density of aqueous cryoprotectant solutions at cryogenic temperatures for optimized cryoprotection and diffraction contrast

Timothy J. Tyree, Ritwik Dan and Robert E. Thorne

S1. Preparation of cryoprotective agent (CPA) solutions.

The cryoprotectants studied were anhydrous methanol from Macron Fine Chemicals; anhydrous ethanol from Decon Labs; 2-propanol from Macron Fine Chemicals; ethylene glycol from Mallinckrodt; glycerol from Fisher Chemical; 2-methyl-2,4-pentanediol (MPD) 99% from Sigma-Aldrich; polyethylene glycol (PEG) 200 from Sigma-Aldrich; and polypropylene glycol (PPG) 425 from Sigma Aldrich.

Cryoprotectant solutions were prepared by combining the desired masses of CPA and distilled deionized water. Masses were measured to an accuracy of ±5 μg using a Mettler Toledo AE240 analytical balance. Solutions were mixed using a Vortex-Genie 2T from Scientific Industries, Inc. until they were optically homogeneous. Drops were generated using 1 mL syringes with needle gauges ranging from 27-33.

Uncertainties in final concentrations were somewhat larger for methanol, ethanol, and 2-propanol solutions, due to their volatility, than for the other CPAs. To minimize concentration errors, monoalcohol solutions were prepared in volumes of ~15 mL and stored in ~15.5 mL test tubes. Syringes for drop dispensing were filled leaving no air space, and their tips capped between measurements.

Table S1 Parameter β in fits of Eq. 5 to data for critical cooling rates (K/s) vs. CPA concentration in % w/v from Warkentin et al., 2013.

**Cryoprotectant**	**β**
ethanol	0.376
methanol	0.302
ethylene glycol	0.206
PEG 200	0.226
glycerol	0.213

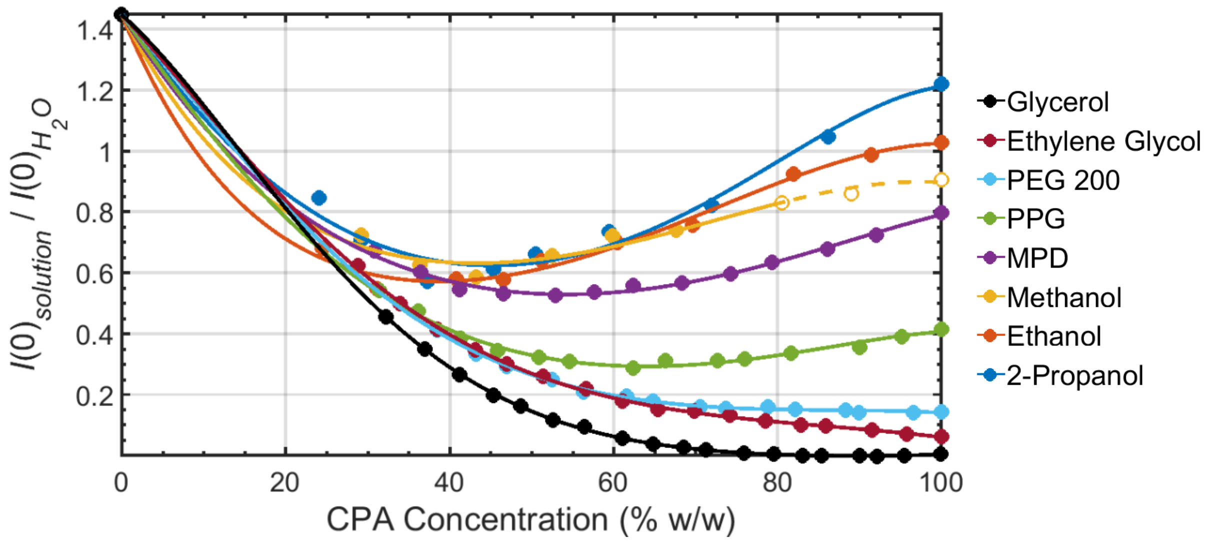

Figure S1 Forward scattering of protein in a CPA solution at T = 77 K (from the present data) normalized by the forward scattering of protein in pure water at T = 300 K. This corresponds to a comparison of signal intensities in cryoSAXS and room temperature SAXS.

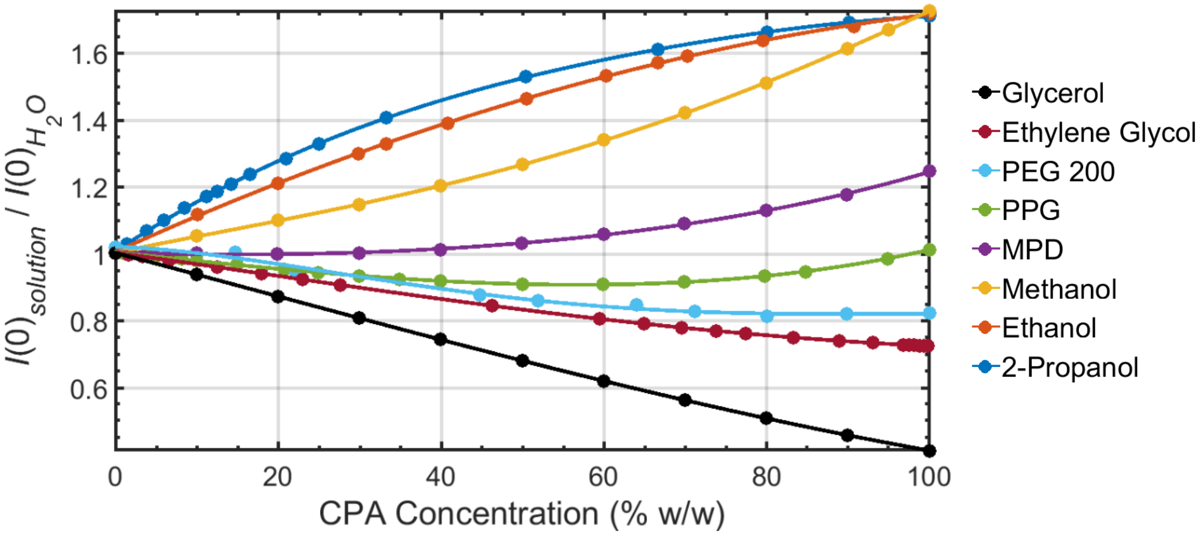

(a)

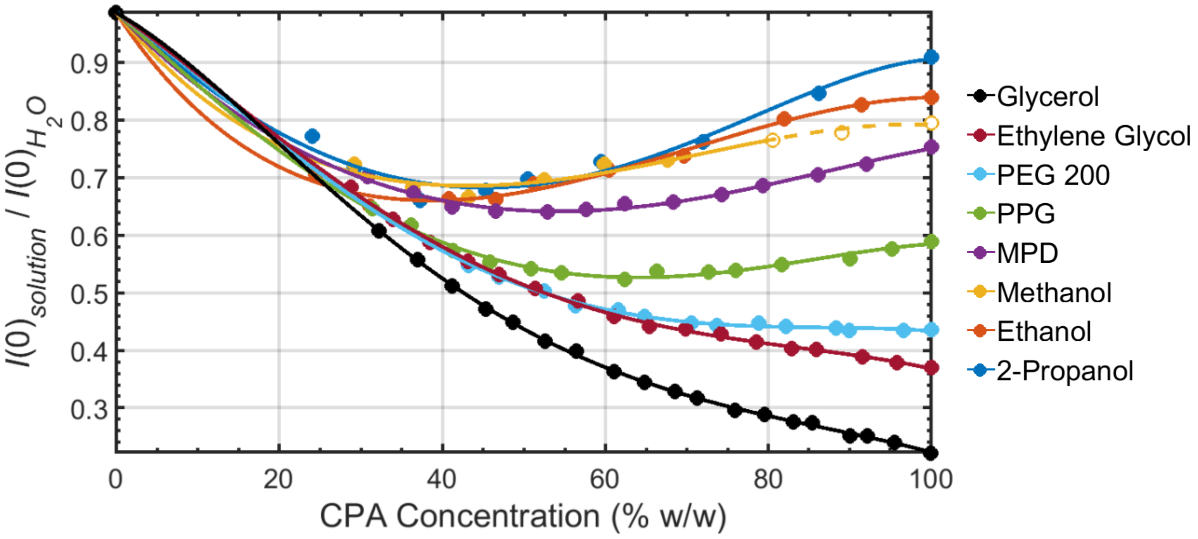

(b)

Figure S2 Forward scattering of protein in a CPA solution (from the present data) normalized by the forward scattering of protein in pure water at (a) T = 300 K and (b) T = 77 K, calculated using Eq. 2, for nucleic acids with $\rho_e \sim 0.55$ $e^- / Å^3$.

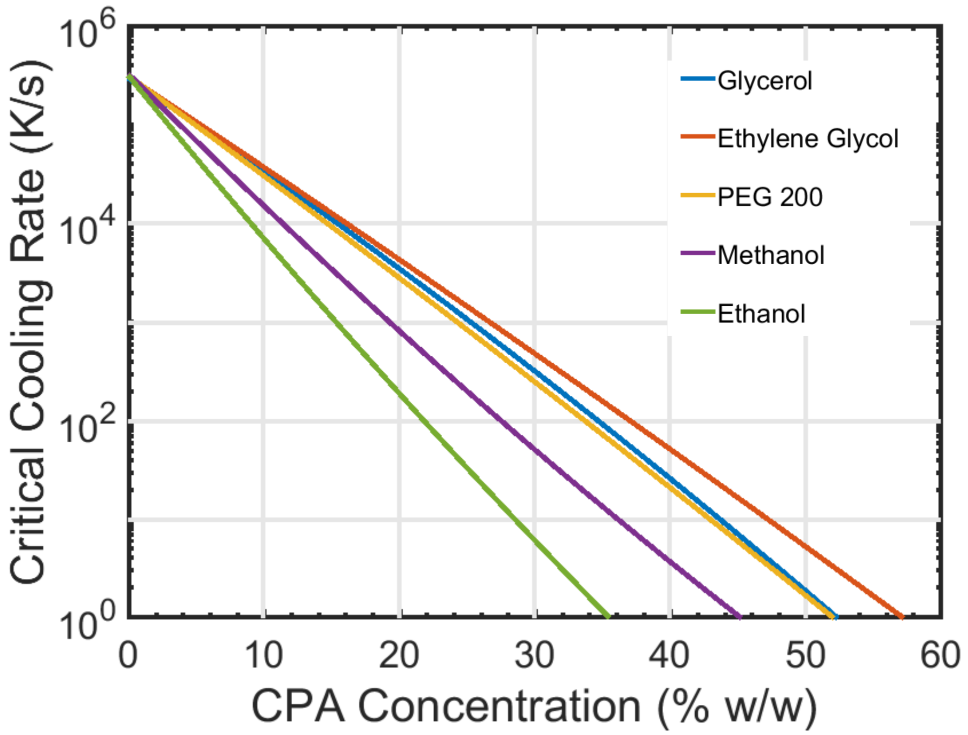

Figure S3 Critical cooling rate (K/s) vs. CPA concentration (% w/w), obtained by combining fits to data for critical cooling rate vs CPA concentration in % w/v (Warkentin, Sethna and Thorne, 2013) of Eq. 5 with parameter β given in Table S1, with fits to previous measurements of the room-temperature densities of each CPA solution shown in Fig. 1 (a) and given in Table 1.

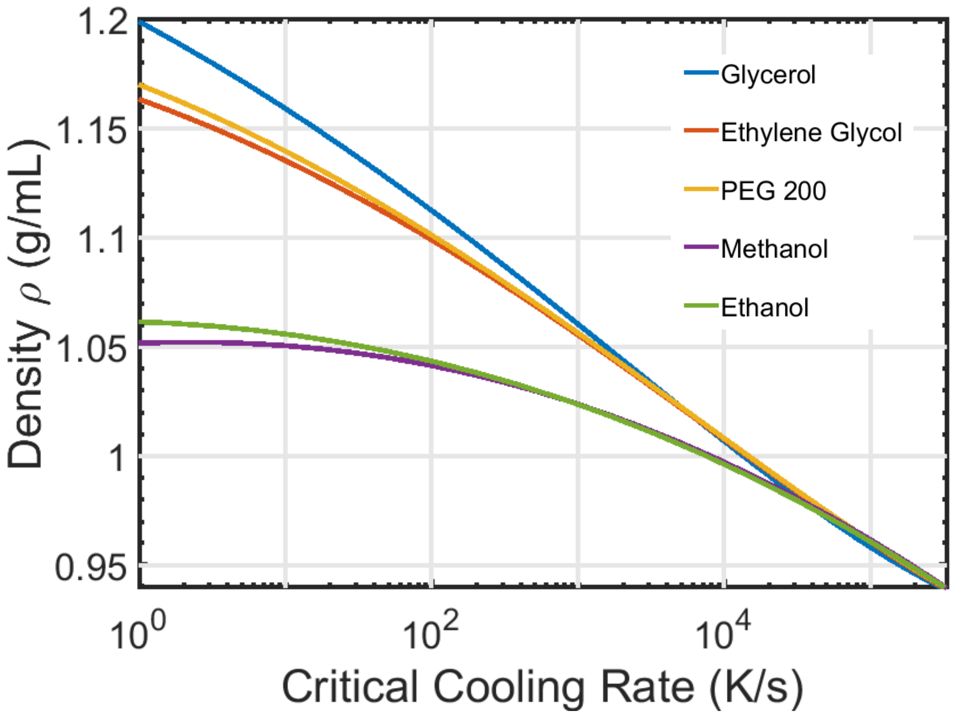

(a)

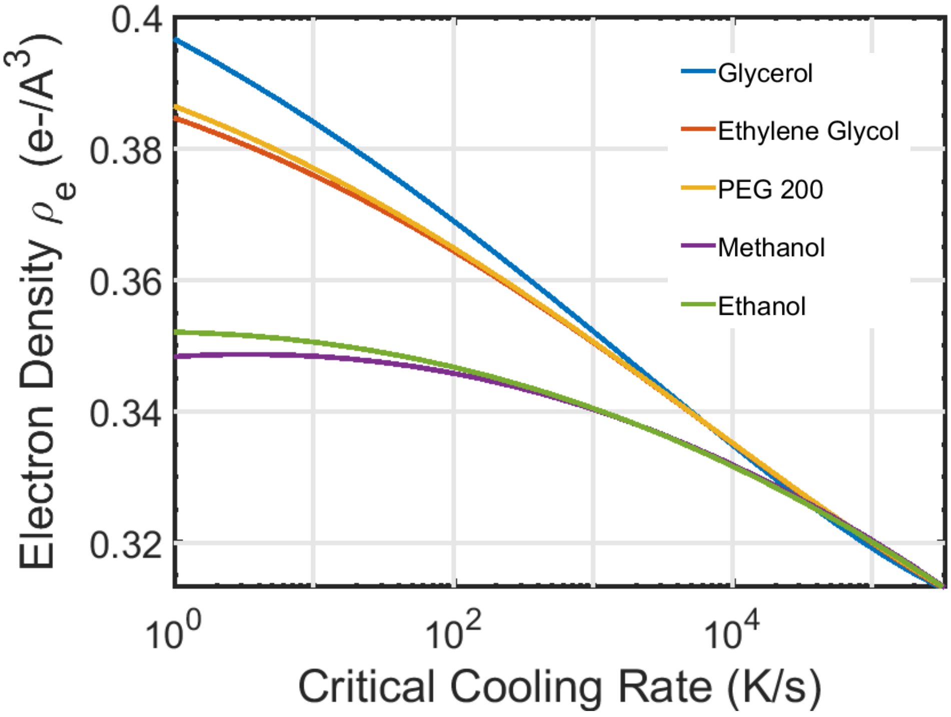

(b)

Figure S4 (a) Density ρ (g/mL) and (b) electron density ρ_e (e^-/Å^3) at T = 77 K vs. critical cooling rate CCR (K/s), obtained by combining fits to the present density data versus CPA concentration in % w/w with fits to CCR versus CPA concentration in % w/w shown in Figure S3.
